# 

**Published:** 1855-04

**Authors:** 


Our next number will close the present volume, and promises to be a good one.

